# Effect of Deepwater Horizon Crude Oil Water Accommodated Fraction on Olfactory Function in the Atlantic Stingray, *Hypanus sabinus*

**DOI:** 10.1038/s41598-018-34140-0

**Published:** 2018-10-25

**Authors:** Eloise J. Cave, Stephen M. Kajiura

**Affiliations:** 1 Department of Biological Sciences, Florida Atlantic University, 777 Glades Road, Boca Raton, FL 33431 USA; 2 Present Address: Department of Ocean Engineering And Marine Sciences, Florida Institute of Technology, 150 West University Blvd, Melbourne, FL 32901 USA

## Abstract

The Deepwater Horizon oil spill was the largest accidental marine oil spill in history, releasing nearly 5 million barrels of crude oil. Crude oil causes both lethal and sublethal effects on marine organisms, and sensory systems have the potential to be strongly affected. Marine fishes rely upon the effective functioning of their sensory systems for detection of prey, mates, and predators. However, despite the obvious importance of sensory systems, the impact of crude oil exposure upon sensory function remains largely unexplored. Here we show that olfactory organ responses to amino acids are significantly depressed in oil exposed stingrays. We found that the response magnitude of the electro-olfactogram (EOG) to 1 mM amino acids decreased by an average of 45.8% after 48 h of exposure to an oil concentration replicating that measured in coastal areas. Additionally, in oil exposed individuals, the EOG response onset was significantly slower, and the clearing time was protracted. This study is the first to employ an electrophysiological assay to demonstrate crude oil impairment of the olfactory system in a marine fish. We show that stingrays inhabiting an area impacted by an oil spill experience reduced olfactory function, which would detrimentally impact fitness, could lead to premature death, and could cause additional cascading effects through lower trophic levels.

## Introduction

Extraction and transportation of oil from offshore reserves creates the potential for the oil to be accidentally released, which could result in significant environmental damage. In 2010, the Deepwater Horizon (DWH) oil spill released nearly 5 million barrels of crude oil into the Gulf of Mexico^1,2^. This massive release of crude oil severely degraded the marine ecosystem immediately surrounding the spill site and directly impacted coastal habitats totaling 1,773 km of shoreline^3^.

Crude oil contains a variety of chemical constituents such as polycyclic aromatic hydrocarbons (PAHs), benzene, toluene, ethylbenzene, xylene, and heavy metals^4,5^. Exposure to hydrocarbons and heavy metals, as well as pesticides and herbicides, is known to damage central or peripheral components of various sensory systems in teleost fishes^6,7^. Known effects include hypersensitivity of and damage to the lateral line^8,9^ metal accumulation in the photoreceptor cells of the eye^10,11^, reduced eye pigmentation^12^, and altered olfactory structure and function^13,14^.

Studies of environmental pollutant impacts upon the olfactory system have focused primarily upon the effect of heavy metals on teleost fishes^6,15–17^. Trace amounts of heavy metals, such as mercury, cadmium, and copper, can detrimentally impact the functioning of olfactory receptor neurons (ORNs) and cause damage or cell death, thus impairing or eliminating olfactory capacity^18–21^. Olfaction is important in all aspects of life history including feeding, and detection of mates and potential predators^22^, and any reduction in olfactory function can reduce fitness.

Despite the obvious importance of olfaction, and the high profile of oil spills, surprisingly few studies have examined the effect of whole crude oil on olfactory function in any marine vertebrate. One study, employing a behavioral assay in a freshwater teleost, suggested that a water-soluble fraction of crude oil impairs olfactory function of Caspian roach, *Rutilus caspicus*^23^. We chose to test the impact of a water-accommodated fraction of crude oil on the olfactory function of a representative elasmobranch fish, the Atlantic stingray, *Hypanus sabinus* (Lesueur, 1824). The elasmobranch fishes (sharks, skates, rays) comprise a diverse subclass that contains many upper- and meso-trophic level predators that exert a key role in preserving marine ecosystem integrity and biodiversity^24,25^. Elasmobranchs possess an olfactory organ that is much larger than comparably sized teleosts, and up to one third of their brain mass is dedicated to olfactory processing^26,27^. Their peripheral olfactory organ (rosette) is overlain with an epithelium that contains microvillous and crypt ORNs, as well as non-sensory cells, including support cells, basal cells, and mucus cells^28,29^. When water-borne chemicals enter the nares, they bind to G-coupled receptor proteins on the ORNs^30^. This results in the activation of the tetrameric cyclic nucleotide gated (CNG) channels on the apical surface of the cell that allow diffusion of Na^+^ and Ca^+^ ions from the seawater into the neuron^31^. An electrode positioned in the seawater immediately above the olfactory epithelium will detect the net negative charge created by the remaining Cl^−^ ions and the output can be recorded as an electro-olfactogram^32^.

We employed an electro-olfactogram technique to quantify the response characteristics of the Atlantic stingray. This benthically-associated, mesotrophic, euryhaline species is abundant in near-shore coastal waters of the western Atlantic, from Chesapeake Bay to Brazil^33^, and is found in the Gulf of Mexico throughout the coastline affected by the DWH oil spill. This species possesses large incurrent and excurrent nares, which facilitate water exchange across the olfactory epithelium and maximize direct contact with the oil-laden seawater. To our knowledge, this study is the first to employ an electro-physiological assay to determine the effects of whole crude oil on the olfactory system of a marine vertebrate.

## Methods

### Preparation

Atlantic stingrays were collected from the Indian River Lagoon, Florida, using a seine net operated by the Florida Fish and Wildlife Conservation Commission, or Florida Atlantic University students. Florida Atlantic University students collected animals in accordance with guidelines approved by the Florida Fish and Wildlife Conservation Commission Special Activities License: SAL-12-1413B-SR. All animals were transported in oxygenated water from the capture location to the Florida Atlantic University Marine Laboratory at the Gumbo Limbo Environmental Complex (Boca Raton, FL, USA). Once at the laboratory, partial water changes were done at approximately 15 minute intervals to gradually acclimate the animals to the ambient water temperature and salinity. The stingrays were then transferred into a 244 × 122 cm fiberglass tank supplied with flow-through seawater from the Atlantic Ocean and covered with a double layer of shade cloth. Stingrays were fed thawed shrimp to satiation every other day and all animals were feeding in captivity for a minimum of one week prior to the start of experimentation.

To determine the toxicological effects of crude oil, stingrays were exposed to a high-energy water accommodated fraction (HEWAF) oil solution. HEWAF stock solution was prepared by blending 4 g of Slick B crude oil in 4 L of seawater (1 g L^−1^) in a 4-L Waring blender (Waring, CB15) for 30 s. An experimental tank, identical to the holding tank (244 × 122 cm), was filled to a depth of 5 cm with seawater (~ 379 L), and 37.9 L of HEWAF stock solution was mixed into the tank. This resulted in a final oil concentration of approximately 0.09 g L^−1^, which was within the range of contamination empirically measured along the Louisiana shoreline after the DWH oil spill (R. Takeshita, ABT Associates). The concentration of total PAHs alone in surface water sampled from the Gulf of Mexico ranged up to 0.146 g L^−1^ ^34^, and PAHs typically account for only 3.9% of the total Macondo oil^35^. For oil exposure treatments, the inflow and outflow valves of the experimental tank were closed, the water was aerated with two air stones, and a stingray was placed into the tank and held in the water containing HEWAF for 48 h. Water quality and oil concentration were maintained with regular water exchanges of 114 L of the HEWAF stock solution every 8 h. For control experiments, stingrays were tested under identical conditions in the holding tank (same aeration, water depth, water change frequency and volume) with no HEWAF added to the seawater.

### Experimental apparatus

Olfactory responses were tested by employing an underwater electro-olfactogram (EOG) to measure summed potentials in the seawater immediately adjacent to the olfactory epithelium^32^. The experimental apparatus was a closed, recirculating, seawater system that consisted of an electrically grounded acrylic experimental tank (89 cm × 43 cm × 21 cm) and an electrically grounded 18.9 L reservoir. An airstone in the reservoir continuously aerated the water and a submersible pump delivered water from the reservoir over the gills of the stingray. A second pump in the reservoir delivered water through an odor delivery tube over the olfactory epithelium. The water in the experimental tank continuously drained back into the reservoir. During control trials the experimental tank and reservoir were filled with untreated seawater and during oil-exposure trials the system was filled with freshly prepared HEWAF oil solution (0.09 g L^−1^ oil concentration). To prevent amino acids and metabolic wastes from accumulating in the system, the entire volume of water in the system was exchanged with untreated seawater, or HEWAF oil solution, every two hours. The EOG experiments could last up to four hours, including setup time, so the total exposure time to HEWAF oil solution could be up to 52 hours by the end of the experiment.

The EOG procedure was similar to that used in previous olfaction studies on elasmobranchs^36–38^. Stingrays were anaesthetized in a solution of 0.1 g L^−1^ tricaine methanesulfonate (MS-222) and, when ventilation ceased, they were injected with 0.5–0.8 mg pancuronium bromide either intramuscularly or intravenously in the caudal vein. Stingrays were then transferred to a submerged platform in the experimental tank and secured ventral side up with Velcro straps. To ventilate the gills, tubes were inserted from the reservoir pump into the spiracles and a sponge was inserted into the mouth, which served to direct water from the spiracles over the gills. A 5 mm diameter odor delivery tube was mounted on a micromanipulator and was positioned with the tip in an incurrent naris with a constant flow of seawater at 3.3 mL s^−1^. Methylene blue dye was injected into the odor delivery tube to confirm adequate flow over the olfactory epithelium.

A Ag-AgCl pellet electrode in an electrode holder (E45P-M15N, Warner Instruments, Hamden, CT, USA) filled with a 3 M KCl solution was used to record olfactory epithelial response to odor stimuli. Fitted to the electrode holder was a 7.5 cm long glass capillary tube (diameter: 1.5 mm) filled with 3 M KCl and 3% agar. The recording electrode was mounted on a micromanipulator and the tip was positioned in the excurrent naris immediately above the olfactory epithelium near the central raphe. An identical reference electrode was secured with the tip of the capillary tube submerged in the seawater in the corner of the tank. Output from the two electrodes was differentially amplified (DP-304, Warner Instruments) at 1,000-10,000x, filtered (HP: 10 Hz, LP: 0.1 kHz, Notch: 50/60 Hz, Humbug, Quest Scientific), digitized at 1 kHz (Power Lab 16/30 model ML 880, AD Instruments), and recorded using LabChart Software (Version 5.5.6, AD Instruments). Because the reference electrode to the differential amplifier was in the grounded seawater, it was also grounded so the recordings were effectively singled-ended.

### Experimental protocol

Olfaction experiments were conducted on seven stingrays under control treatments and eight stingrays under oil treatments. Five amino acids were chosen that represented each amino acid group (Cysteine-polar, Alanine-non-polar, Phenylalanine-neutral/aromatic, Glutamic acid-acidic, and Arginine-basic) and had been previously demonstrated to be stimulatory to this species^36^. Stock solutions of the amino acids were freshly made the day before each experiment and stored at 4 °C. Amino acids used in HEWAF treated stingrays were dissolved in the HEWAF solution whereas amino acids used in control treatments were dissolved in untreated seawater. At the start of an experimental trial, a 1 mV calibration pulse from the amplifier was recorded and subsequently used to calibrate the magnitude of the responses. Test stimuli (0.5 mL) were injected into the latex tubing immediately above the odor delivery pipette and the continuous water flow transported the injected bolus over the olfactory epithelium. The resultant response elicited from the olfactory receptor neurons was detected by the recording electrode. Test stimuli included a seawater procedural control (either untreated or HEWAF-treated), a 1mM L-Alanine standard, and the other four test amino acids in random order. Each stimulus was delivered three minutes after the previous injection, which allowed the EOG trace to return to its approximate pre-injection baseline level (estimated visually). Each series of amino acids was tested three times, and the responses averaged for each individual.

The LabChart software was used to measure three parameters that described the shape of the EOG response to amino acid stimuli; the magnitude, duration, and slope. The magnitude (μV) was defined as the maximum voltage displacement from the baseline after a stimulus injection. The duration (s) was defined as the time from the onset of the polarizing response to the time when the response began to return to baseline. The slope (μV s^−1^) was defined as the maximum change in voltage during the initial polarizing response immediately after the amino acid injection. Amino acid responses were compared within each treatment group (untreated or oil-treated seawater) using a one-way ANOVA followed by a Bonferroni corrections *post hoc* test (α = 0.05) for paired comparisons between amino acids and the seawater control. The mean response magnitudes for each amino acid were tested between control and oil exposed stingrays using a one-way ANOVA (α = 0.05). A log_10_ transformation was applied to EOG magnitude response values to meet assumptions of normality and homoscedasticity. The EOG response durations were tested using a non-parametric Mann-Whitney U test (α = 0.05) because the data did not meet the assumptions of normality and homoscedasticity even after log_10_ transformation. The slopes of the responses were tested using a one-way ANOVA (α = 0.05). All statistical analyses were conducted using SPSS (Version 24, IBM).

All animal care and experimental procedures conformed to the guidelines established by the Florida Atlantic University Institutional Animal Care and Use Committee protocol: A13–21.

## Results

Electro-olfactogram responses to 1 mM concentrations of five highly stimulatory amino acids^36^ and a seawater control were recorded from the olfactory epithelium of stingrays maintained under untreated seawater control (n = 7) and oil exposure (n = 8) treatments. For each stingray, the response magnitude, duration, and initial response slope, were quantified for each amino acid.

### Response Magnitude

**Figure 1:**
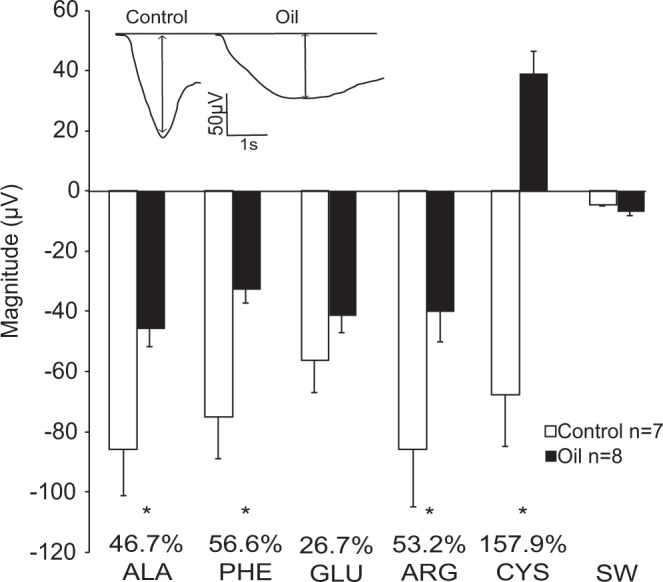
Mean (+s.e.) EOG response magnitudes (μV) of *Hypanus sabinus* to five highly stimulatory amino acids and a seawater control (SW). Negative values represent EOG negative polarizing responses. Percentages represent the percent change between control responses and oil exposed responses. A representative EOG response is shown to indicate the measured parameter. Ala, alanine; Phe, phenylalanine; Glu, glutamic acid; Cys, cysteine; Arg, arginine. * indicates significant differences between control and oil exposed animals for each amino acid (one-way ANOVA, p < 0.05).

Response magnitude (μV) was averaged among all individuals of a given treatment for each amino acid (Fig. 1). The mean EOG response magnitude did not differ significantly among all of the amino acids within either the control or oil exposure treatments (ANOVA; Control: F = 33.258, P = 0.002; Oil: F = 27.624, P < 0.001; Bonferroni *t-*test; P < 0.001 for all amino acid comparisons). However, all amino acids elicited a significantly greater response than the seawater control. The magnitude of the EOG responses to the L-isomers of alanine, phenylalanine, cysteine, and arginine in oil-exposed stingrays was significantly less than that from the untreated seawater control treatment (ANOVA; Alanine, F = 7.24, P = 0.019; Phenylalanine, F = 10.75, P = 0.006; Arginine, F = 6.74, P = 0.022; Cysteine, F = 12.38,P = 0.004). The response magnitude to glutamic acid did not differ significantly between control and oil-exposed stingrays (ANOVA; F = 1.53, P = 0.236). Fig. 1 shows representative traces of the response to 1 mM alanine from control and oil treatments. The response magnitude was consistently smaller in oil-exposed animals, with declines of 26.67% to 157.85% of the values for control animals. The response to glutamic acid was reduced by 26.67%, followed by alanine (46.72%), arginine (53.19%), phenylalanine (56.62%), and cysteine (157.85%). In oil-exposed animals tested with cysteine, the response consistently exhibited a positive slope, which differed from all other amino acid treatments. This percent reduction is expressed as the difference between the peak of the positive response in oil-exposed animals and the negative response in control animals. A subset of individuals was tested with L-methionine, which also contains a sulfur group like cysteine, but methionine did not elicit the same positive response.

### Response Duration

**Figure 2:**
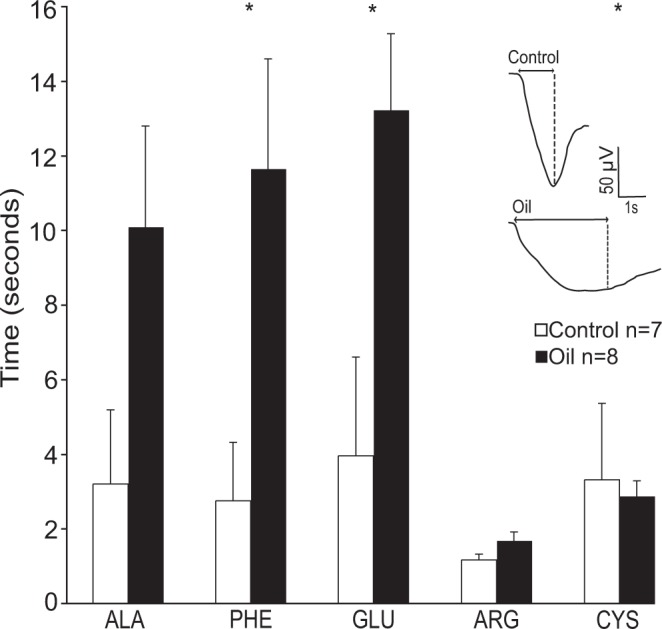
Mean (+s.e.) EOG response duration (seconds) in *Hypanus sabinus* from five highly stimulatory amino acids. A representative EOG response is shown to indicate the measured parameter. Ala, alanine; Phe, phenylalanine; Glu, glutamic acid; Cys, cysteine; Arg, arginine. * indicates significant differences between control and oil exposed animals for each amino acid (Mann-Whitney U, p < 0.05).

Response duration (s), from the onset of the response to the maximum deflection, was measured from each EOG response (Fig. 2). The response duration in oil-exposed animals always matched or exceeded the duration for control animals. The response duration was significantly longer in oil-exposed animals for the amino acids phenylalanine, glutamic acid, and cysteine (Mann-Whitney U; Phenylalanine, U = 7.00, P = 0.015; Glutamic Acid, U = 11.00, P = 0.049; Cysteine, U = 10.00, P = 0.037). In contrast, response duration did not differ between oil exposed and control animals for alanine and arginine (Mann-Whitney U; Alanine, U = 12.00, P = 0.064; Arginine, U = 16.00, P = 0.165).

### Initial Response Slope

**Figure 3:**
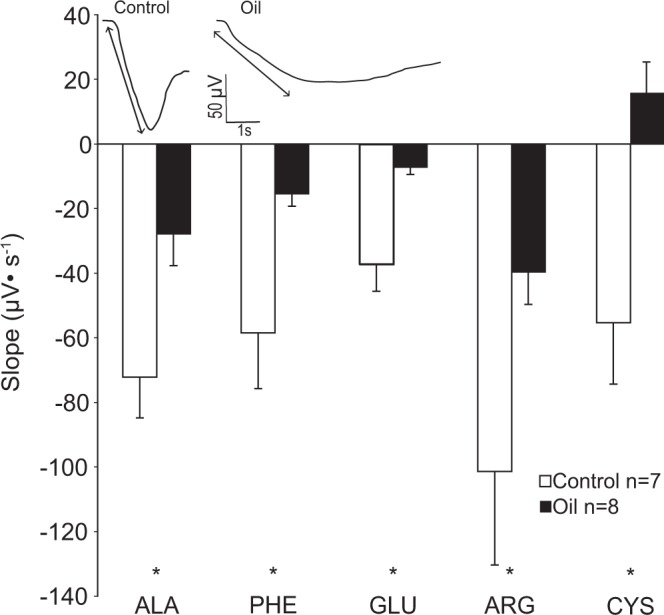
Mean (+s.e.) slopes (μV•s^−1^) of EOG response magnitudes in *Hypanus sabinus* from five highly stimulatory amino acids. A representative EOG response is shown to indicate the measured parameter. Ala, alanine; Phe, phenylalanine; Glu, glutamic acid; Cys, cysteine; Arg, arginine. * indicates significant differences between control and oil exposed animals for each amino acid (one-way ANOVA, p < 0.05).

The slope of the initial change in potential (μV s^−1^) was measured from each EOG response (Fig. 3). The initial response slope differed significantly between control and oil treatments for all amino acids (ANOVA; Alanine, F = 7.91, P = 0.015; Phenylalanine, F = 8.05, P = 0.014; Glutamic Acid, F = 13.00, P = 0.003; Arginine, F = 4.856, P = 0.046; Cysteine, F = 7.14, P = 0.019). Stingrays tested under control treatments exhibited a significantly greater negative slope than stingrays exposed to oil treatments. Cysteine consistently elicited a negative slope under control treatments and a positive slope under oil treatments. This was the only amino acid to generate a change in sign of the slope. The slope in control animals ranged from 2.5 to 5.2 times greater than the slope in oil-exposed animals. The much steeper slope in control animals indicates a more rapid response onset to amino acid stimuli.

## Discussion

This study is the first to quantify the physiological effects of whole crude oil on the olfactory function of a marine vertebrate. Elasmobranchs are renowned for their well-developed sensory systems, which are critical to inform them about the presence of predators, prey, mates, and unfavorable environmental conditions. Any impairment of these sensory systems could have a detrimental effect on their survival and fitness. This study determined that exposure to crude oil, at concentrations empirically measured in coastal areas following the DWH oil spill, significantly impaired olfactory function in the Atlantic stingray, *H. sabinus*. Response magnitude, duration, and onset, were all significantly different in stingrays exposed to crude oil compared to stingrays that were held in untreated seawater conditions. Unlike other sensory systems in which the receptor cells are not in immediate contact with the environment (eye, inner ear, lateral line, electroreceptors), the chemo-sensory cells of the olfactory organ are directly exposed, through the mucus, to the seawater. As a result, environmental pollutants have the ability to directly damage the receptor cells and affect olfactory function. In this study, the observed physiological response dynamics included (i) a reduced response magnitude, (ii) a protracted response duration, and (iii) a reduced initial response slope. There are three general hypotheses that could account for these results.

The observed results could be explained by a diffusion barrier, which would impede the transport of odorants between the seawater environment and the olfactory epithelium. The crude oil might act as a physical barrier through which the odorants must diffuse, or the oil could irritate the olfactory epithelium and stimulate an increase in mucus production, which would provide a similar diffusion barrier. The increased diffusion distance could increase the diffusion time through the barrier, and reduce the number of odor molecules that reach the receptor sites. This would result in a reduced initial response slope and a reduced response magnitude. As a result, the odor molecules would take longer to bind to and disassociate from the receptor sites, thus keeping ion channels open for longer than normal resulting in a protracted response duration.

The functional role of the mucus layer is to provide a protective surface on the olfactory epithelium through which odorants must diffuse to reach the ORN’s^39^. Increased mucus production would increase the diffusion distance to the ORN’s^13^. The olfactory epithelium produces considerable amounts of mucus in response to hydrocarbon contamination^40^. Mucus production in the olfactory epithelium is regulated by acetylcholine (ACh) secretion^41^. Several anthropogenic toxicants, such as pesticides and insecticides, inhibit ACh activity, which results in increased mucus production and a concomitant reduction of responsiveness of ORNs to natural olfactory stimuli^42^. Therefore, an increase in mucus production by inhibition of ACh or irritation to the olfactory epithelium could explain the significantly altered EOG response magnitude, duration and delayed initial onset.

Another explanation for the observed results could be the constituents of the crude oil binding or coupling with the amino acid odorants as they are transported in the water that enters the olfactory capsule. This could result in a conformational change that detrimentally impacts binding specificity to the receptor sites, which would effectively mask the odorant from the receptors^43^. Similarly, the crude oil constituents might competitively bind to the receptor sites directly, effectively blocking them from being activated by the presence of an amino acid odorant. However, crude oil is not ionic, whereas amino acids are zwitterions, so the possibility of an interaction between them is remote. Likewise, the receptor sites would also require charged amino acid residues to successfully bind, so again, the crude oil might not be capable of competitive binding.

Finally, the observed results could be realized if the crude oil constituents resulted in receptor cell damage or death. Cell death, loss of cilia and or microvilli (teleosts), or loss of microvilli (elasmobranchs), will reduce the capacity for odor molecules to bind to receptor sites and thus compromise the capability to initiate a physiological response. Reduced EOG responses and cellular damage due to various chemical pollutants have been previously documented, but only a handful of studies have examined the effects of crude oil on the olfactory epithelium^40,44,45^. Crude oil, at concentrations less than 0.51 mg L^−1^, caused a loss of cilia on the olfactory epithelium of pink salmon^45^. Similarly, tidewater silversides, *Menidia beryllina*, exposed to 5% water-soluble fraction (WSF) crude oil for 7 days exhibited sensory cell death of the olfactory epithelium^40^. The same study found similar cell death in the hogchoker, *Trinectes maculatus*, at higher concentrations of 50% WSF crude oil. Fathead minnows, *Pimephales promelas*, exposed to WSF aviation fuel for 72 hours exhibited cell necrosis in the olfactory rosette^46^. Similarly, exposure of small killifish, *Fundulus heteroclitus*, to naphthalene resulted in loss of olfactory sensory cilia^47^. If naphthalene, a component of crude oil, caused damage to the olfactory cilia in a teleost, then it is likely that it would also damage the dendritic knobs on the microvillous cells in elasmobranchs. Additionally, water-soluble fractions of crude oil have caused hyperplasia, necrosis, and lesions on the olfactory epithelium^44^. All of these physical insults may result in reduced olfactory sensitivity to chemical stimuli.

The proposed mechanisms to explain the observed results are not mutually exclusive and could act synergistically to impair olfactory function. Regardless of the mechanism, significant alteration of the olfactory response was observed with a relatively high concentration (1 mM) of amino acid stimulus. A deliberately high concentration was chosen because it would yield a large magnitude response. A large response facilitates quantification of reduction in response magnitude due to oil exposure. Although it is unlikely that animals would encounter stimuli at such a high concentration in nature, a similar reduction in EOG response would probably also be manifest at biologically relevant sub-micromolar concentrations. This impairment of olfactory function could reduce the ability to detect prey, predators, and mates, thus resulting in a detrimental impact upon fitness. A decrease in the fitness of the organism could contribute to a population level decline, leading to cascading effects through lower trophic levels creating ecological imbalances^48,49^.

The oil exposed stingrays were maintained and tested entirely in HEWAF treated seawater, with amino acids dissolved in the same concentration of HEWAF treated seawater. This was done to simulate the conditions to which they would be exposed in nature in the event of an oil spill, and the concentration used mimicked that found in nearshore waters following the DWH spill. However, the physical nature of the HEWAF treated seawater itself might be responsible for some of the observed responses. If the HEWAF treated seawater were more viscous, for example, it could take longer to contact the molecular receptors and to be cleared from the olfactory organ after stimulation. This could account for the slower onset and increased duration of the EOG response from the oil treatment animals. Physiological impairments due to oil exposure, such as a loss of microvilli, would persist after removal from the HEWAF treatment solution. Therefore, it would be informative to test oil exposed animals in “clean” seawater to see if the same response reductions are present. This would help to tease apart whether observed differences in response were due to physical characteristics of the solvent, or due to physiological effects from HEWAF exposure.

Of particular interest is the consistent and anomalous response to cysteine. Cysteine was the only amino acid that generated a positive EOG response in oil exposed stingrays and a normal negative EOG response in stingrays that were exposed to untreated seawater conditions. This phenomenon has not been documented previously in either teleosts or elasmobranchs but it has been documented in frogs^50^, newts^51^, and tortoises^52^. One example that has caused a positive EOG is when barium ions and a degenerated epithelium caused the blocking of chloride ions from entering the cell but not potassium ions from exiting the cell^50,52^. It remains unclear whether the observed peripheral physiological response results in central processing of the cysteine in the normal manner.

In this study, crude oil produced similar reductions in the EOG magnitude as those seen in teleost fishes when exposed to environmental pollutants^18,53–55^. Crude oil contains many complex organic and inorganic compounds, including heavy metals such as aluminum, manganese, cobalt, copper, zinc, and mercury^4^. Heavy metals can block sodium and calcium ion channels in the olfactory systems of teleosts, resulting in reduced olfactory responses^56^. Copper causes a concentration-dependent decrease in EOG responses in coho salmon, *Oncorhynchus kisutch*, thus reducing the sensitivity to both bile salts (taurocholic acid) and amino acid mixtures (L-serine)^57^. Mercuric chloride (HgCl_2_) exposure also reduces the EOG response amplitudes to various amino acids in Atlantic salmon, *Salmo salar*^55^. A similar study with Atlantic salmon found that exposure to 10^−5^ M mercury reduced the EOG responses to L-alanine by 35% of the pre-exposure response^54^. This suggests that inhibition of amino acid response by mercury is mediated at the level of the olfactory receptor^17^.

Although this study focused upon a shallow water, coastal species, deep-water elasmobranch species may be highly susceptible to crude oil exposure. It is estimated that up to 10 million gallons of crude oil from the DWH remain in the sediment at the bottom of the Gulf ^58^ and may be more toxic than that found at the surface, which could cause more severe physiological damages, including impairment of sensory systems. In particular, deep-sea benthic species like skates, which develop for prolonged periods in egg cases on the seafloor, could be continuously exposed to high concentrations of crude oil in the sediment throughout sensitive developmental periods. Also, because the metabolic rate of marine organisms declines significantly with temperature, and hence depth^59^, deep-sea elasmobranch species have a much slower metabolic rate than shallow water species^60,61^ and thus might metabolize crude oil much more slowly. This prolonged exposure could manifest as different or more severe results. Also, under field conditions, animals are likely to encounter variable exposure concentrations, which may be higher or lower than the concentration used in this study. This acute exposure has the potential to induce other physiological responses, potentially compounding the adverse effects of the altered olfactory function. Even if the oil does not cause immediate or direct mortality, sub-lethal effects could still reduce fitness or contribute to premature death.

## Data Availability

The datasets generated during and analyzed during the current study are available from the corresponding author on reasonable request.
